# Reply to “Reconsidering Cost‐Effectiveness Claims of Short‐Term Prevention Services in Japan”

**DOI:** 10.1111/ggi.70191

**Published:** 2025-10-02

**Authors:** Ryota Watanabe, Masashige Saito, Kazushige Ide, Katsunori Kondo

**Affiliations:** ^1^ Faculty of Health and Medical Sciences Aichi Shukutoku University Nagakute Aichi Japan; ^2^ Center for Well‐Being and Society Nihon Fukushi University Nagoya Aichi Japan; ^3^ Faculty of Social Welfare Nihon Fukushi University Chita‐Gun Aichi Japan; ^4^ Center for Preventive Medical Sciences Chiba University Chiba Chiba Japan; ^5^ The Japan Agency for Gerontological Evaluation Study Kashiwa Chiba Japan

To the Editor,

We thank Riaz and colleagues [1] for their thoughtful comments on our article [2], “Difference in long‐term care cost obtained with the short‐term intensive prevention service (day service type C): A 3‐year follow‐up study of Japanese older adults.” In this reply, we clarify four points raised in their letter.

First, on international evidence, Riaz and colleagues cite a Dutch cluster‐randomized trial [3] that evaluated a staff reablement training program against usual care without training, with outcomes and costs assessed from a societal perspective. Our study instead compared participants versus non‐participants of the short‐term intensive prevention service and evaluated public long‐term care (LTC) cost within Japan's LTC insurance framework [4]. Because the intervention, comparator, analytic perspective, and institutional context differ, a head‐to‐head comparison is not appropriate. Notably, prior studies [5, 6] also report cost reductions with reablement‐type services, which is not inconsistent with our finding.

Second, regarding selection bias and unmeasured confounding, as pointed out in their letter, we agree that non‐randomized comparisons are subject to unmeasured confounding. We therefore used propensity score–based inverse probability weighting with regression adjustment and adjusted for baseline functional and sociodemographic measures; results were consistent over 3 years. In addition, a Japanese randomized reablement trial [7] reported non‐use of LTC insurance services at 3 months in 11.1% versus 3.8% (intervention versus control), which is consistent with our interpretation. The cancer study [8] cited in their letter illustrates that propensity score–matching improves balance on measured covariates; it does not claim that propensity methods ‘often fail’ to capture uptake–utilization dynamics.

Third, concerning social participation, the letter cites evidence [9] linking social participation to lower cumulative LTC costs. Our study of short‐term intensive prevention service was explicitly designed to support continued social participation after the 3‐month program; thus, social participation is an intended program, not a competing explanation. As reported in our article [2], 80% of participants engaged in social participation after short‐term intensive prevention service. Enabling sustained participation is a plausible mechanism by which costs are moderated.

Fourth, on temporal confounding and regression to the mean, baseline functional status differed between groups as shown in Table 1 of our original article [2], and we adjusted for these differences. If regression to the mean were the primary driver, larger cost declines would be expected in the group with higher baseline functional‐decline risk; instead, we observed a sustained difference favoring participants. We also examined results by the fiscal year of program initiation and obtained similar findings. These checks make it unlikely that regression to the mean and concurrent policy changes explain the observed cost differences.

Within the constraints of an observational design, our analyses provide real‐world evidence from Japan's LTC insurance system that a short‐term prevention service may contribute to more appropriate LTC costs. We appreciate the critique by Riaz and colleagues and hope these clarifications are useful.

## Disclosure

This study was conducted by the Japan Agency for Gerontological Evaluation Study and the Center for Well‐being and Society, Nihon Fukushi University, with funding and commission from the Omron Corporation.

## Ethics Statement

Ethical approval was obtained from Nihon Fukushi University (23‐028‐02), Chiba University (M10460), and the Japan Agency for Gerontological Evaluation Study (2019‐01).

## Data Availability

No new datasets were generated for this letter. The analyses relied on the same dataset as the original article. Owing to ethical and contractual restrictions, the data are not publicly available.
